# Do People “Pop Out”?

**DOI:** 10.1371/journal.pone.0139618

**Published:** 2015-10-06

**Authors:** Katja M. Mayer, Quoc C. Vuong, Ian M. Thornton

**Affiliations:** 1 Institute of Neuroscience, Newcastle University, Newcastle upon Tyne, United Kingdom; 2 Neural Mechanisms of Human Communication, Max Plank Institute for Human Cognitive and Brain Sciences, Leipzig, Germany; 3 Department of Cognitive Science, Faculty of Media & Knowledge Sciences, University of Malta, Msida, Malta; Centre de Neuroscience Cognitive, FRANCE

## Abstract

The human body is a highly familiar and socially very important object. Does this mean that the human body has a special status with respect to visual attention? In the current paper we tested whether people in natural scenes attract attention and “pop out” or, alternatively, are at least searched for more efficiently than targets of another category (machines). Observers in our study searched a visual array for dynamic or static scenes containing humans amidst scenes containing machines and vice versa. The arrays consisted of 2, 4, 6 or 8 scenes arranged in a circular array, with targets being present or absent. Search times increased with set size for dynamic and static human and machine targets, arguing against pop out. However, search for human targets was more efficient than for machine targets as indicated by shallower search slopes for human targets. Eye tracking further revealed that observers made more first fixations to human than to machine targets and that their on-target fixation durations were shorter for human compared to machine targets. In summary, our results suggest that searching for people in natural scenes is more efficient than searching for other categories even though people do not pop out.

## Introduction

As both actor and observer, we have a lifetime of experience with the human body [1]. Perceptually, we encounter hundreds of bodies every week “in the flesh” and through media such as television and the internet. The purpose of the current study was to establish whether the nature of human bodies such as their distinct physical form and movements in combination with our vast experience with them, has specific consequences for visual attention. To answer this question, we used a visual search paradigm in which observers searched natural scenes for human targets performing a wide variety of actions embedded in an array of mechanical distractors and vice versa. In this way we measured how efficiently observers search for people relative to another category with very different shapes and movements.

Previous research has shown that movement, in particular, plays an important role for processing people (see [2], for a review). The most direct demonstration of this is that observers can extract a rich variety of information from point-light actors in which only the movements of key body parts like the head, elbows, knees, and feet are visible [3]. Although this sensitivity depends to some extent on the global configuration of the human body (e.g., [4]), Troje and Westhoff [5] speculated that there may be a “life detector” mechanism that quickly and reliably detects ballistic movements of local parts (e.g., the movement of the feet) which signals the presence of other animate beings. Observers also automatically process the motion of other humans to the extent that this processing can interfere with other tasks [6–8] and can lead to reflexive attentional orienting [9]. Furthermore, human motion affects how children and adults allocate visual attention to social scenes [10].

Movements of the human body are thought to be processed at a range of levels within the visual system [11] via a distributed network of brain regions [12–14]. The superior temporal sulcus (STS) is a primary structure within this network [12].

Apart from motion, the static global form of the human body clearly plays an important role in processing people as well [4], [15–18]. In particular, recent evidence suggests that static depictions of the human body can automatically attract the eyes and attention [15], [19] and may even have preferential access to consciousness [20]. As with body motion, body form appears to be processed in a variety of brain areas, with much current research focusing on occipitotemporal body-selective regions (see [21], for a review), specifically the extrastriate body area (EBA; [22], [23]) and the fusiform body area (FBA; [24], [25]).

Recent evidence further suggests that these two aspects of person perception—human motion and characteristic human body form—may be functionally as well as neurally dissociable [26]. However, regardless of whether they operate alone or together in a given task, they clearly have the potential to efficiently solve what has been termed the basic problem for the “social brain”, namely, “is there someone else there?” [23]. To our knowledge, no previous study has applied standard visual search methodology to directly examine efficiency when human figures with all form and motion information available need to be detected in natural scenes. In our study, we use visual search tasks with natural scenes containing humans or machines to investigate whether human body form and human movement allow better detection of humans with respect to machines.

How efficiently observers can search a visual array can be quantified by a slope that relates the time to find a target to the number of items in the array [27], [28]. Highly efficient “pop-out” search would be characterized by a slope close to zero; that is, search times would be independent of the number of items to be searched. Search slopes greater than zero would indicate lower degrees of search efficiency. Pop-out search has been demonstrated for basic features such as colour and orientation [27]. With respect to complex visual stimuli, there is evidence that static faces can pop-out [29] but this finding remains contraversial [30], [31]. Although, as previously mentioned, there is evidence that human body form can capture attention [15] this has not been demonstrated within the context of visual search tasks, so it is unclear whether these stimuli pop out.

Search tasks have been applied to biological motion stimuli such as point-light walkers displaying human or animal movements, but to date, no pop-out search has been demonstrated (e.g., [32], [33], [34]). Similarly, it is also effortful to search for particular human actions (e.g., boxing) amidst other distractor actions (e.g., marching; [33]). However, it was shown that search for familiar human movements shown as point-light displays can be more efficient than for unfamiliar non-biological motion [32], [34]. This research indicates that i) search for humans may be more efficient than search for other natural objects and that ii) human movements may play a key role for the efficient search performance.

What might be the underlying mechanisms for efficient visual search? Both “bottom-up” (e.g., [5]) and “top-down” (e.g., [8], [14], [32], [35], [36–38]) mechanisms could contribute to efficient search for moving people. For example, Troje and Westhoff’s [5] “life detector” could operate in a bottom-up manner, treating local ballistic motion as a basic feature much like colour to facilitate search [34]. To explain the better efficiency searching for familiar movements, Cavanagh et al. [32] suggested that “attentional sprites” could be deployed in a top-down manner to familiar movements in the environment, efficiently guiding search behaviour. Cavanagh et al. had observers search for left facing walkers (composed of 11 points) amidst right facing ones (or vice versa) and found that search efficiency was approximately 120 ms/item. A second group of observers search for an unfamiliar, and arguably simpler, 2-point tumbling motion amidst 2-point orbiting motions (or vice versa). Observers’ search efficiency for these stimuli increased dramatically to approximately 600–700 ms/item. Presumably, observers’ experience with people has built up sprites that can efficiently search for familiar human movements whereas they have no such sprites for the unfamiliar tumbling/orbiting motion.

In the search studies concerned with attentional sprites and life detectors, point-light walkers were used to bypass several challenges faced by the visual system in natural scenes such as segmentation. Thus, these findings could be over-estimating our ability to detect people in real-world scenes. On the other hand, the distractors were often other human bodies (left/right facing walkers: [32], different human actions: [33], upright/inverted walkers: [34]), which may shed little light on the basic question of detection. Similarly, the great reduction in static form information with point-light stimuli removes one important route to person detection, and so may be serioulsy under-estimating person detection ability. In the current work, we therefore attempted to test the generality of earlier findings by using natural scenes and by contrasting searches for humans relative to machines.

Observers in our study searched for humans amidst distractor scenes containing machines and vice versa. As human bodies occur in motion and standing still in the natural environment we included both search arrays with dynamic (Experiment 1) and static (Experiment 2) scenes. Importantly, the static condition allowed us to measure the contribution of form information per se (e.g., configuration of parts). We previously demonstrated a dynamic advantage for detecting humans in natural scenes [38] and that dynamic point-light walkers but not static ones interfere with processing a target walker [8]. Based on previous work and our own studies, we predict more efficient search for humans relative to machines even when targets are presented in natural scenes. Furthermore, we predict more efficient searches for dynamic relative to static humans. We also measured eye movements during visual search. This allowed us to measure how much time is needed to process a target using fixation duration as a proxy, how quickly a target can draw the eyes towards it using the latency from the onset of the search array to a fixation landing on a target as a proxy, and the likelihood that targets attract observers’ first saccade in the search arrays [39]. Thus, by supplementing search slopes with fixation parameters, we can better understand the strategies underlying search for dynamic and static humans in natural scenes.

## Methods

### Participants

Sixteen participants recruited from the wider Newcastle University community completed the experiments either in return for course credit or on a voluntary basis (14 females, age: 18–25 yrs). They were new to the task and naïve to the research goals. Prior to the experiments, participants were instructed and gave written informed consent. Eight participants were tested with video clips (Experiment 1) and the other 8 were tested with static images from the video clips (Experiment 2). Experiments were conducted in accordance with the Declaration of Helsinki. Ethics approval was provided by the ethics committee of Newcastle University. The individuals shown in our figures gave written informed consent to be visible in the figures.

### Stimuli and Apparatus

**Fig 1:**
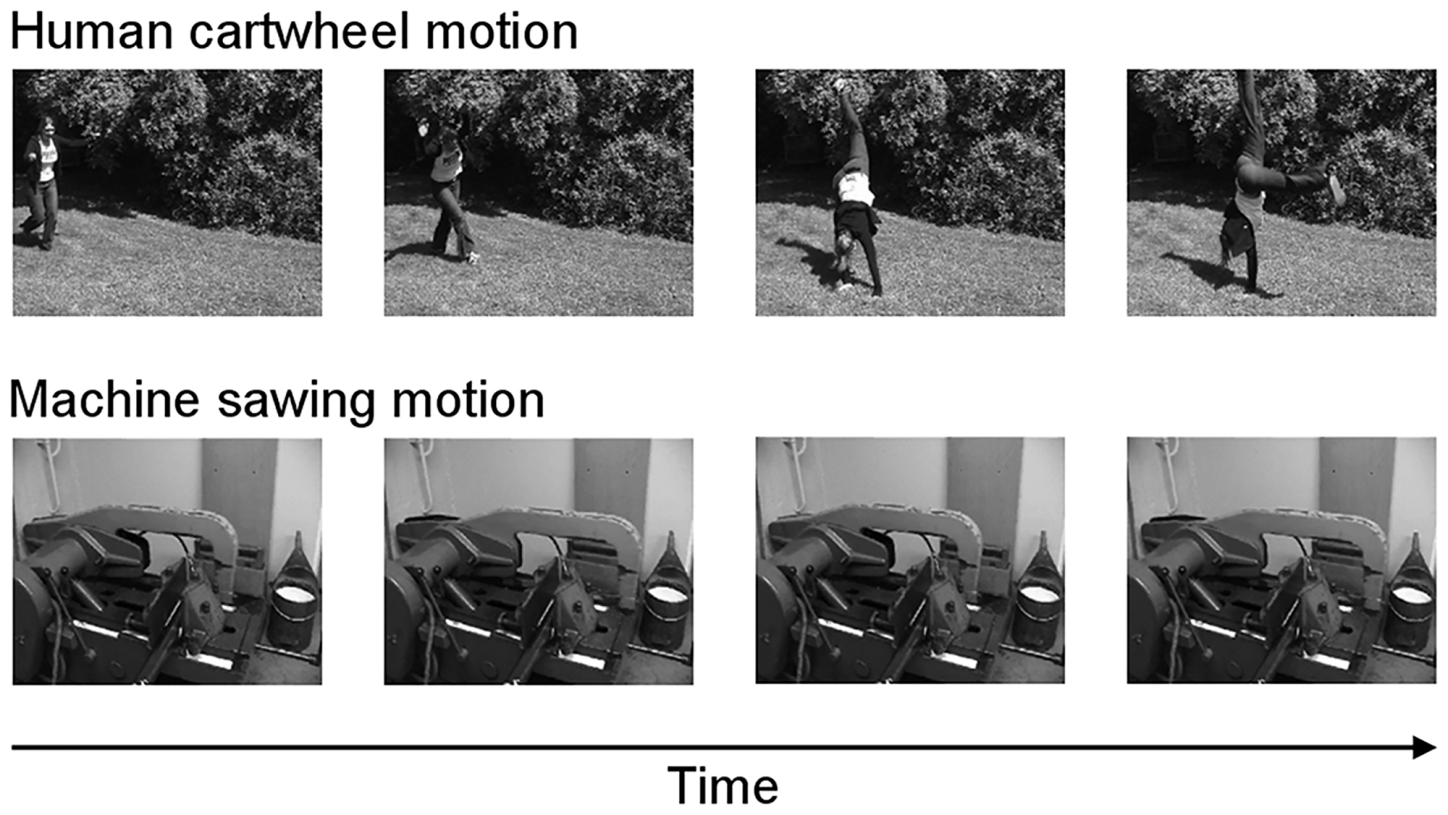
Stimuli. Top-row: Frames taken from a video displaying human motion. Bottom-row: Frames taken from a video showing mechanical motion.

The stimuli consisted of 1.8 s video clips. There were two target categories of clips: human motions and mechanical motions, with 8 scenes in each category. The human motions consisted of a person performing a cartwheel, washing dishes or walking down the stairs for example. The mechanical motions consisted of a sawing machine, a truck unloading stones or a carousel for example. Each scene contained a single predominant object type from the target category and there were never any objects from the other category. Stimuli were taken from films and documentaries or acquired with a camcorder. Frames from two example videos are shown in Fig 1. The videos had a frame rate of 25 frames per second and frames were 128 pixel x 96 pixel greyscale images.

Participants viewed the stimuli from a distance of 50 cm. At this distance, the stimuli subtended 5.4° x 4.1° visual angle. They were seated at a Cambridge Research System eyetracker, which tracked at 50 Hz with a spatial resolution of 0.1°. The experiment was controlled by a Windows PC, running Matlab and the psychtoolbox [40], [41]. A Sony Trinitron CRT monitor was used to present the stimuli. It had a refresh rate of 100 Hz and a resolution of 1024 pixel x 768 pixel. Head motions were constrained by a chin rest.

### Design and procedure

Participants searched for a scene from the target category (i.e., human or machine) amidst scenes from the other category. The target was either absent or present and the search arrays varied in number of items to be searched. Thus, there were 3 within-subject factors: target type (human, machine), trial type (target present, target absent) and set size (2, 4, 6, and 8 scenes in the search array). The presentation of the target category was blocked, and the order was counterbalanced across participants.

Prior to the experiments, participants were presented with all scenes to ensure that they were familiar with the target and distractor scenes. They first saw all 8 scenes from the target category in a 2 rows x 4 columns array, and wrote a brief description of each scene on a piece of paper. Afterwards, they saw all the scenes from the distractor category and carried out the same procedure. The scenes were shown as videos (Experiment 1) or a randomly chosen frame from the videos (Experiment 2). The familiarization phase took approximately 5 min.

**Fig 2:**
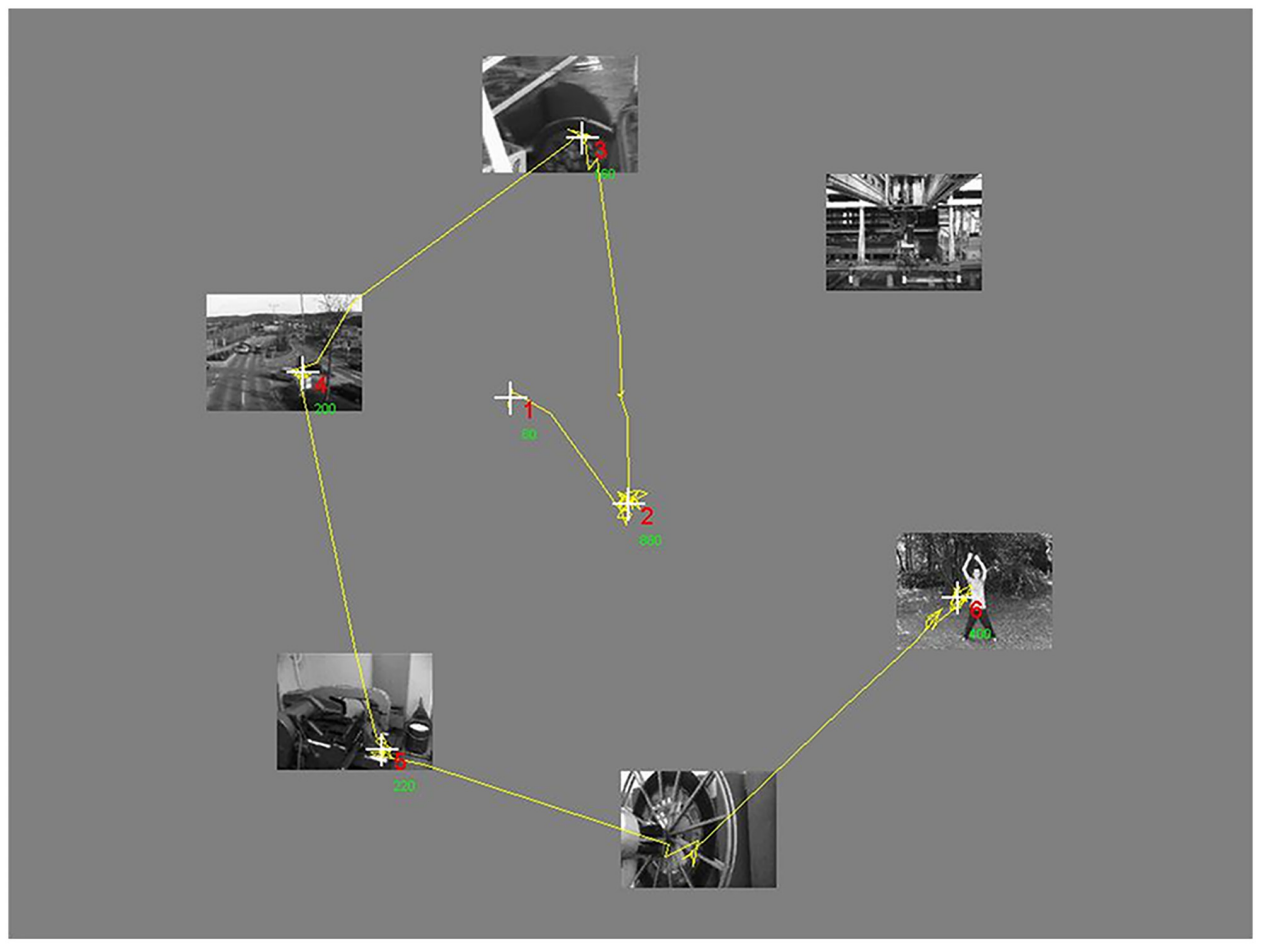
Search array. Yellow line: eye movements when searching the array. White crosses: fixations. Red numbers index the order of the fixations. Green numbers are fixation durations in milliseconds.

For the experiments, we used circular search arrays in which the center of each video/image was placed on an invisible circle with a radius of 300 pixels (12.5°) from the center of the screen. The scenes were evenly distributed around this circle. The starting orientation for placing each scene was randomly determined from trial to trial (with 0° being the top of the screen). Fig 2 provides an example search array, along with the eye-movement trace of a representative participant for a single trial.

Each trial began with a white fixation cross at the center of a grey screen. The search array was presented 1000 ms after fixation onset and remained on the screen until participants responded. The fixation cross remained visible throughout the trial. The videos used in Experiment 1 started on a randomly selected frame on each trial, and they were played in a continuous loop. The images used in Experiment 2 were frames randomly sampled from each video on each trial. Participants pressed the ‘c’ or ‘m’ key as quickly and as accurately as possible to indicate whether a scene from the target category was present or absent. The response mapping was counterbalanced across participants. If participants responded incorrectly, there was a 1500 Hz feedback tone. There was a 500 ms blank grey screen following the response before the next trial began. We started tracking the right eye from the onset of the fixation.

There were a total of 256 trials for each target block. These trials were divided into 4 sets of 64 trials. On each set, the 8 scenes from the target category were shown once at each set size on present trials. The distractors were randomly sampled from the 8 distractor scenes. On absent trials, the stimuli in the search array were randomly sampled from the 8 distractor scenes. There was an equal number of present and absent trials. The trials within each set were randomly presented. There was a self-timed break after each set. We calibrated the eye tracker at the beginning of each target category block. The entire experiment took about 40 min.

### Data analysis

The search time was measured from the onset of the search array to the time participants responded. The search slope was computed by linear regression of the median correct search time onto set size. We used the median search time to control for outliers. Accuracy in all experiments was high (> 80%). Search slopes fitted to the accuracy showed a significant effect of trial type on the accuracy slope for static images (*F*(1, 7) = 13.08, *p* = .009, $\eta_{p}^{2}$ = .65): present trials had a small negative slope averaged across participants (*M* = -0.4% per scene, *SE* = 0.2% per scene) whereas absent trials had a small positive slope averaged across participants (*M* = 0.1% per scene, *SE* = 0.2% per scene). No other main effects or interactions were significant (*p*s > .36).

For the eye-movement data, we analyzed fixation duration, fixation latency and proportion of fixations from correct trials. We focused our eye-movement analyses on “first” fixations [39]. This means that, on present trials, the fixation duration was measured for the first fixation that landed on the target scene in the search array on each trial. On absent trials, the fixation duration was measured for the first fixation that landed on any scene in the array on each trial. To be considered on a scene, fixations needed to be within 100 pixels (4.1°) of the centre of that scene. The latency of the first fixation was the duration between the onset of the search array and the onset of the first fixation on the target. The proportion of first fixations was computed as the number of first fixations that landed on a target divided by all first fixations that landed on any scene in the search array for all trials within a given condition. The last two measures can only be computed for present trials.

Custom software was used to extract fixations from each trial. The raw gaze time series were smoothed using a median filter within a moving 60 ms temporal window. Blinks were removed. All gaze points that were within 0.6° of each other and within least a 120 ms temporal window of each other were counted as a single fixation. We further restricted our fixations to those from trials in which the eye tracker successfully tracked the eye for 70% or more of the trial duration. For most participants in Experiments 1 and 2, this criterion removed between 1.2% and 10.3% of the fixation data. The criterion removed 51.2% of the fixation data for one participant and 17.6% for another (both from Experiment 1).

To limit biases in our results due to saliency differences between human and machine videos and images, we trained model observers on the search task and compared their search behaviour to the participants’ search behaviour.

## Results

### Dynamic natural scenes: Experiment 1 (videos)

**Fig 3:**
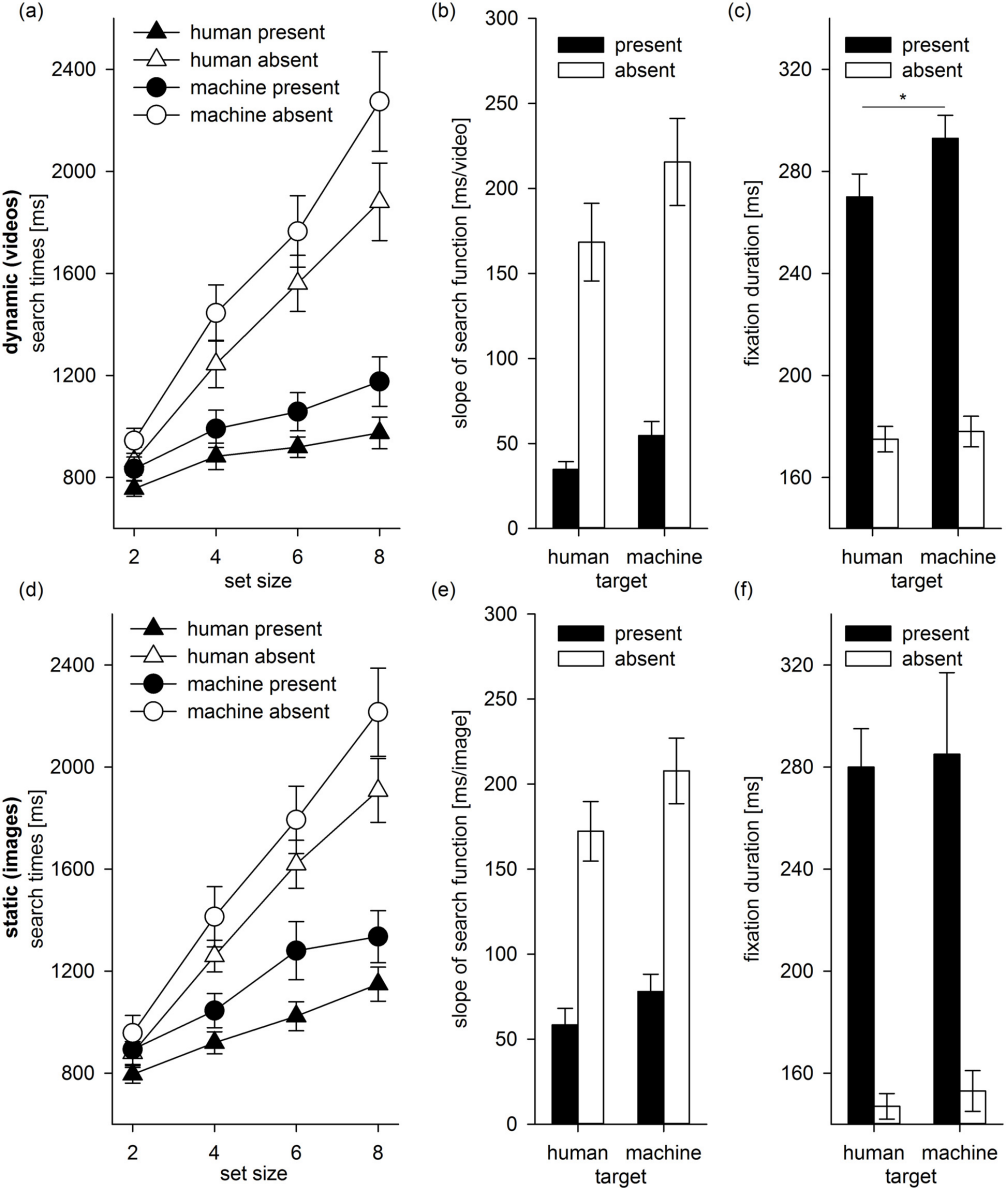
Results. (a)–(c): Results of Experiment 1 (dynamic natural scenes). (d)–(f): Results of Experiment 2 (static natural scenes). Error bars are +/-1 standard error of the mean.

Fig 3a–3c show the median search times as a function of set size, the search slopes fitted to the search times, and the fixation durations averaged across participants for the different conditions in Experiment 1. We focused our analyses on search slopes and fixation parameters. A 2 target type (human, machine) x 2 trial type (absent, present) repeated measures ANOVA revealed that search slopes (Fig 3b) were steeper for machine compared to human targets (*F*(1, 7) = 12.91, *p* = .01, $\eta_{p}^{2}$ = .65) and steeper for absent compared to present trials (*F*(1, 7) = 60.68, *p* < .001, $\eta_{p}^{2}$ = .90). There was no interaction between target type and trial type (*p* = .15). Search slopes were significantly greater than zero in all conditions (1-sample *t*-tests, all *p*s < .001). This indicates that search times increase with set size, even when participants search for dynamic human targets (Fig 3a).

Fig 3c shows the mean first fixation duration for humans and machines on present and absent trials. Table 1 presents the data for the fixation parameters as a function of target type and set size for present trials. A 2 (target type) x 2 (trial type) x 4 (set size) repeated measures ANOVA on the fixation durations revealed that fixation durations were shorter for human compared to machine targets (*F*(1, 7) = 6.08, *p* = .04, $\eta_{p}^{2}$ = .47), and shorter for absent compared to present trials (*F*(1, 7) = 142.11, *p* < .001, $\eta_{p}^{2}$ = .95). Target type and trial type interacted (*F*(1, 7) = 7.99, *p* = .03, $\eta_{p}^{2}$ = .53): fixation durations were shorter for humans than machines on present trials (paired-samples *t*-test, *t*(7) = 2.83, *p* = .03) but not on absent trials (paired-samples *t*-test, *t*(7) < 1). There was a marginal effect of set size (*F*(3, 21) = 2.65, *p* = .08, $\eta_{p}^{2}$ = .27; Table 1), and no other interactions reached significance (*p*s > .14).

**Table 1:** Fixation parameters for Experiment 1 (videos).

	Fixation Duration		Fixation Latency		Proportion First	
Set Size	M [ms]	SE [ms]	M [ms]	SE [ms]	M	SE
Humans						
2	270	11	379	17	.81	.04
4	271	15	461	15	.67	.07
6	249	16	527	26	.62	.04
8	275	25	565	23	.58	.06
Machines						
2	299	10	362	12	.73	.04
4	295	19	478	38	.57	.07
6	294	19	492	9	.49	.04
8	303	36	486	22	.39	.05

M: mean; SE: standard error of the mean. Fixation Latency: time between the onset of the search array and the onset of the first fixation on target. Proportion First: proportion of first fixations on target. All values refer to target present trials.

For fixation latency, a 2 (target type) x 4 (set size) ANOVA showed no main effect of target type (*p* = .14; Humans: *M* = 483 ms, *SE* = 18 ms; Machines: *M* = 454 ms, *SE* = 14 ms). There was a main effect of set size (*F*(3, 21) = 36.61, *p* < .001, $\eta_{p}^{2}$ = .84) and a marginal interaction between target type and set size (*F*(3, 21) = 3.02, *p* = .052, $\eta_{p}^{2}$ = .30; Table 1). A post-hoc test showed that fixation latency differed between human and machine targets only for set size 8, *t*(7) = 3.44, *p* = .01 (all other comparisons, *p*s > .13; Table 1).

Lastly, a 2 (target type) x 4 (set size) ANOVA on the proportion of first fixations on present trials revealed a main effect of target type (*F*(1, 7) = 13.86, *p* = .007, $\eta_{p}^{2}$ = .66) which indicated that a larger proportion of first fixations landed on human compared to machine targets (Humans: *M* = .67, *SE* = .04; Machines: *M* = .55, *SE* = .05). Furthermore, there was a main effect of set size (*F*(3, 21) = 25.16, *p* < .001, $\eta_{p}^{2}$ = .78) indicating that the proportion of first fixations on target decreased with set size (Table 1). Target type and set size did not interact (*p* = .21).

### Static natural scenes: Experiment 2 (images)

Fig 3d–3f show the median search times as a function of set size, the search slopes, and the fixation durations averaged across participants for the different conditions in Experiment 2. A repeated measures ANOVA revealed that search slopes (Fig 3e) were steeper for machine targets compared to human targets (*F*(1, 7) = 12.61, *p* = .01, $\eta_{p}^{2}$ = .64), and steeper for absent trials compared to present trials (*F*(1, 7) = 47.12, *p* < .001, $\eta_{p}^{2}$ = .87). Target type and trial type did not interact (*p* = .17). Search slopes were significantly greater than zero in all conditions (1-sample *t*-tests, all *p*s < .001). This indicates that search times increase with set size when participants search for static human targets (Fig 3d).

Table 2 presents the data for the fixation parameters as a function of target type and set size for present trials in Experiment 2. For fixation duration, a 2 (target type) x 2 (trial type) x 4 (set size) repeated measures ANOVA revealed main effects of trial type (*F*(1, 7) = 72.73, *p* < .001, $\eta_{p}^{2}$ = . 91), and set size (*F*(3, 21) = 6.78, *p* = .002, $\eta_{p}^{2}$ = .49). Fixation durations were shorter on absent compared to present trials and they decreased with increasing set size (Table 2). In contrast to Experiment 1, there was no difference between human and machine targets for fixation duration (*p* = .70; Fig 3f). Trial type interacted with set size (*F*(3, 21) = 3.24, *p* = .04, $\eta_{p}^{2}$ = .32): the decrease in fixation duration with set size was larger on absent trials compared to present trials (Table 2). No other interactions reached significance (*p*s > .35).

**Table 2:** Fixation parameters for Experiment 2 (images).

	Fixation Duration		Fixation Latency		Proportion First	
Set Size	M [ms]	SE [ms]	M [ms]	SE [ms]	M	SE
Humans						
2	286	21	372	15	.70	.03
4	294	18	444	21	.66	.04
6	255	12	502	14	.52	.08
8	286	20	532	23	.43	.07
Machines						
2	294	32	351	9	.69	.07
4	306	50	427	26	.46	.06
6	290	44	480	19	.42	.05
8	249	23	583	31	.38	.04

M: mean; SE: standard error of the mean. Fixation Latency: time between the onset of the search array and the onset of the first fixation on target. Proportion First: proportion of first fixations on target. All values refer to target present trials.

As with dynamic stimuli, a 2 (target type) x 4 (set size) ANOVA for fixation latency showed no main effect of target type (*F* < 1.0; Humans: *M* = 462 ms, *SE* = 9 ms; Machines: *M* = 460 ms, *SE* = 19 ms). There was a main effect of set size (*F*(3, 21) = 41.63, *p* < .001, $\eta_{p}^{2}$ = .86) and a significant interaction between target type and set size (*F*(3, 21) = 3.49, *p* = .03, $\eta_{p}^{2}$ = .33; Table 2). A paired-samples *t*-test showed that the difference between the two target types was only significant for set size 2 (*t*(7) = 2.94, *p* = .02; all other *p*s > .19).

Lastly, we analyzed the proportions of first fixations on target with a 2 (target type) x 4 (set size) repeated measures ANOVA. There was a main effect of target type (*F*(1, 7) = 13.64, *p* = .008, $\eta_{p}^{2}$ = .66): participants made significantly more first fixations to human targets than to machine targets on present trials (Humans: *M* = .58, *SE* = .04; Machines: *M* = .49, *SE* = .05). There was also a main effect of set size (*F*(3, 21) = 26.60, *p* < .001, $\eta_{p}^{2}$ = .79) indicating that the proportion of first fixations that landed on the target decreased with increasing set size (Table 2). Target type and set size did not interact (*p* = .12).

There is evidence that motion facilitates scene processing [38]. We submitted search slopes fitted to the search times on present trials to a 2 (experiment: dynamic, static) x 2 (target type: humans, machines) mixed design ANOVA to further test whether motion may be more efficiently used for humans compared to machines [32], [34]. There were main effects of experiment (*F*(1, 14) = 4.81, *p* = .046, $\eta_{p}^{2}$ = .26) and target type, (*F*(1, 14) = 13.80, *p* = .002, $\eta_{p}^{2}$ = .50). Participants were more efficient searching for dynamic (*M* = 44.73, *SE* = 6.14) compared to static targets (*M* = 68.21, *SE* = 8.77), and they were more efficient searching for human (*M* = 46.57, *SE* = 6.01) compared to machine targets (*M* = 66.37, *SE* = 7.00). However, there was no significant interaction between experiment and target type, *F* < 1.0, indicating that motion equally facilitated search for human and machine targets. We also submitted the fixation parameters averaged across set size to a similar mixed design ANOVA. For fixation duration and latency, there were no significant main effects or interactions (*p*s > .17). For the proportion of first fixations on target, there was only a main effect of target type (*F*(1, 14) = 26.81, *p* < .001, $\eta_{p}^{2}$ = .66; *p*s > .19 for all other effects): the proportion of first fixations was higher for human (*M* = 0.62, *SE* = 0.03) compared to machine targets (*M* = 0.52, *SE* = 0.03).

### Saliency model observer

Overall, the results suggest that participants process human targets more efficiently than machine targets. With natural scenes, it can be difficult to rule out stimulus differences between human and machine targets that could affect how efficiently scenes are processed independently of their category membership [42], [43]. To explore the extent to which image differences between human and machine targets could lead to different search performance for the two target categories, we simulated artificial model observers who searched for a target based on a saliency map which indicates visually important locations in an image [44], [45]. These visually important, or salient, locations are based on different image features. Briefly, a saliency map is computed by first sampling an image (in our case, the entire visual array composed of individual scenes) at several spatial scales for different image features to create a set of feature maps. These feature maps are then normalized and linearly summed into a single saliency map. For videos, the features we used included image intensity (6 scales), local image orientation (6 scales; 4 orientations: 0°, 45°, 90° and 135°), and local motion energy (6 scales); for static images, these features included only image intensity and local image orientation.

To simulate search behaviour, each model observer sequentially “fixates” locations in a saliency map, starting with the location that has the highest salience and followed by locations with progressively lower salience. A winner-take-all algorithm is used to select a salient location for each fixation which allowed us to measure the number of cycles (i.e., search time) to select a winning location. We terminate the search when the model fixates on the target item; that is, the model observer did not have to perform any object detection which is beyond the scope of this study. In this manner each model observer can scan a visual array searching for the target.

The saliency maps in the current simulations were computed using the Saliency Toolbox (version 2.1; [46]). We used default values from the toolbox to compute image intensity and local image orientation feature maps. To compute the local motion energy feature map, we transformed each video into its equivalent motion energy video. For each original video, we take sequential pairs of frames (1–2, 2–3, …) and compute optic flow using the Lucas-Kanade algorithm [47]. The algorithm estimates the displacement of each pixel from Frame *N* to Frame *N* + 1, and provides a vector indicating the direction and magnitude of the motion displacement with subpixel resolution. For simplicity we used the magnitude rather than the motion energy in different directions (e.g., [48]). The magnitude images were saved as frames into a motion energy video. We used the Lucas-Kanade algorithm implemented in Piotr Dollar’s Image and Video Matlab Toolbox (http://vision.ucsd.edu/~pdollar/toolbox/doc/index.html).

We tested 8 model observers with dynamic visual arrays and 8 with static arrays. We always presented arrays with a target present. Each model was tested in a target type (human and machine) x set size (2, 4, 6, and 8) design. There were 20 repetitions for each condition. For each repetition, the target and distractors items were randomly selected to create the visual array. Thus, although the computation of the saliency map is completely deterministic for a given visual array, the randomization of scene selection on each trial introduced variability into the model observers’ search behaviour. The model observer’s performance in each condition was determined by the number of cycles it took to “fixate” on any location within the target image in the array. With dynamic visual arrays, we first randomly selected the start frame for each video and the corresponding frame in the motion energy video. We then computed the saliency map and the time for the model to select a winning location. The next frame used to compute the subsequent saliency map was based on this duration. The videos were looped if the selected frame went beyond the total number of frames.

As with human observers, we computed search slopes for human and machine targets. For our model observers tested with dynamic visual arrays, there was no significant difference in search slopes between human and machine targets with dynamic visual arrays (paired-samples *t*-test, *t*(7) = 1.67, *p* = .14; Humans: *M* = 89 cycles/scene, *SE* = 9 cycles/scene; Machines: *M* = 112 cycles/scene, *SE* = 11 cycles/scene). There was also no significant difference in search slopes between target types for model observers tested with static arrays (paired-samples *t*-test, *t*(7) = 0.12, *p* = .91; Humans: *M* = 94 cycles/scene, *SE* = 13 cycles/scene; Machines: *M* = 96 cycles/scene, *SE* = 11 cycles/scene).

## Discussion

In the present study, we used a visual search paradigm to investigate whether human bodies automatically attract attention and pop out when embedded in search arrays containing mechanical distractors. Despite the high level of relevance of human bodies for social interaction, we did not find evidence for pop-out processing of dynamic or static human targets in natural scenes. First, search times for both human and machine targets increased as a function of set size. Second, the latency for first fixations to land on a target was the same for human and machine targets (although there were some differences between target categories depending on the number of distractors). Although humans do not pop out in natural scenes, observers were more efficient at searching for human compared to machine targets. For both static and dynamic scenes, observers found human targets more quickly than machine targets, they were less influenced by the number of distractors for human targets (i.e., shallower slope), and they made more first fixations to human targets. Observers also fixated more briefly on dynamic human targets than on dynamic machine targets, whereas no such difference was found with static targets. Taken together, observers’ search and eye-movement behaviours suggest that there may be some form of tuning of the visual system to more efficiently process human form and motion compared to mechanical form and motion even though people do not appear to pop out.

Several previous studies using point-light displays have found processing advantages for biological motion compared to non-biological motion (e.g., [5], [9], [32], [34], [49]). Our results extend these findings to natural scenes. However, not all studies have found an advantage for biological motion over non-biological motion with point-light displays, at least for detection. Hiris [42], for example, found that detection performance for point-light walkers was no better than detection of non-biological motion, provided that the non-biological motion was structured (e.g., a translating rectangle defined by dots). Thirkettle et al. [43] found that the performance on a point-light walker detection task declined when the distance between the dots marking the ankles of the point-light walker declined. This finding implied that biological motion per se is not sufficient for rapid detection.

Findings such as Hiris’ [42] or Thirkettle et al.’s [43] suggest that the rapid detection of biological motion reported in previous studies may depend on the precise viewing conditions provided in the experiment. Therefore, it is important to match biological and non-biological motion stimuli for adequate comparisons of performance when processing the two types of stimuli. In our study, we aimed to provide scenes displaying a rich environment which limited how well we could match the videos containing humans and machines. To minimize any effects of stimulus differences between the two scene categories, we included a wide range of actions for both categories, used greyscale images, and we familiarized observers with all scenes prior to the experiments. We further simulated model observers that searched for dynamic and static human and machine targets in the search arrays based on saliency computed from image intensity, local image orientation and local image motion energy (for videos) feature maps [44]-[46], [48]. The model observers’ did not show more efficient search for human than machine targets. Though not definitive, this finding helps to rule out the possibility that image differences between the two target categories lead to human observers’ more efficient search for people compared to machines at least for the simple features tested here.

In addition to these controls, other factors beyond the simple “biological/non-biological” distinction could also influence search behaviour in the current work. First, as pointed out in the opening to this paper, we have a great deal more experience with human bodies than with almost any other type of object. Although we carefully familiarised participants with the videos from both categories, it seems likely that our more general expertise in perceiving and controlling bodies could also contribute to the observed efficiency advantage. Second, mechanical actions were mostly selected from different machine categories whose exemplars, consequently, had quite large differences in shape. Our biological actions, in contrast, were all from the same basic category (i.e., human body) with comparably small shape differences. This difference in category heterogeneity could conceivably aid top-down target selection and could, at least in part, account for the observed search asymmetries.

Previous studies have used point-light displays because they afford a high degree of stimulus control [42], [43]. For these stimuli, there is evidence that both bottom-up life detectors [5] and top-down sprites [32] underlie efficient search for biological motion [32], [34]. Our results suggest that both types of mechanisms may also underlie efficient search behaviours for dynamic humans in natural scenes. Our comparisons between dynamic and static scenes further indicate that motion facilitated search for both human and machines. That we found a search advantage for static human compared to static machine targets, however, suggests that the human form does contribute to efficient search for humans. Taken together with previous findings, our study suggests that observers can use either form or motion to search for humans in natural scenes more efficiently than other categories (e.g., machines, see [11]). Future studies will need to explore the interaction between these and other possible bottom-up and top-down mechanisms in the perception of biological motion and static human bodies under more natural viewing conditions.

## Conclusion

Although people do not pop out in natural scenes, observers are more efficient at processing humans compared to machines. This efficiency was reflected in both search times and eye fixation patterns. Our results suggest that searching for humans, though more efficient than other categories, remains effortful and requires attentional resources (e.g., [32], [34]). These results help to generalize previous findings with point-light displays to natural scenes.
